# Establishment of patient-derived tumor spheroids for non-small cell lung cancer

**DOI:** 10.1371/journal.pone.0194016

**Published:** 2018-03-15

**Authors:** Zengli Zhang, Huiqian Wang, Qifeng Ding, Yufei Xing, Zhonghua Xu, Chun Lu, Dongdong Luo, Longjiang Xu, Wei Xia, Caicun Zhou, Minhua Shi

**Affiliations:** 1 Department of Respiration, The Second Affiliated Hospital of Soochow University, Suzhou, P.R. China; 2 Invitrocue Biomedical Service Suzhou, Suzhou, P.R. China; 3 Department of Thoracic & Cardiac Surgery, The Second Affiliated Hospital of Soochow University, Suzhou, P.R. China; 4 Department of Pathology, The Second Affiliated Hospital of Soochow University, Suzhou, P.R. China; 5 Department of Medical Oncology, Shanghai Pulmonary Hospital, Thoracic Cancer Institute, Tongji University School of Medicine, Shanghai, P.R. China; University of North Carolina at Chapel Hill School of Medicine, UNITED STATES

## Abstract

The prognosis of advanced non-small cell lung cancer (NSCLC) patients is poor. One of the reasons for this hampered progress has been a lack of *in vitro* models that would faithfully recapitulate the heterogeneity of tumors and response to treatment. In this study, surgically resected tumors were obtained from patients with stage I/II NSCLC during curative-intent surgery. Using a 3D patient-derived tumor spheroids culture system, our results demonstrate successful long-term expansion of primary NSCLC cells in vitro (> 120 days). Patient-derived tumor spheroid (PDS) cultures could be established with a success rate of 100% (3 out of 3 samples). Consistent with their growth in culture and their cancer type, many cells within the tumor spheroids were stained positive for Ki67 and thyroid transcription factor-1. The result of this study supports the establishment of an expandable 3D *in vitro* NSCLC model for drug screening, and enables the potential long term studies such as the establishment of drug resistant models.

## 1 Introduction

Lung cancer has been the leading cause of cancer death in the world for several decades. Although considerable improvements have been made in recent years, the prognosis of NSCLC patients remains poor with a five-year survival rate of only 1–5 percent. This is partly due to the use of established lung cancer cell lines by most pharmaceutical companies for the first-pass screening of large libraries of compounds. These cells are usually cultured in traditional two-dimensional (2D) monolayers on flat and rigid 2D plastic substrata which do not reflect actual characteristics present in most patients’ tumors [1]. They often lack cell-cell and cell-extracellular matrix interactions required for maintaining their cellular functions and defining cell phenotypes. More importantly, these cell lines lack cancer cell heterogeneity as the most undifferentiated cells would outgrow other cells under the selection pressure exerted by the artificial *in vitro* environment [2]. To circumvent some of the issues with the use of cell lines and their lack of clinical relevance, there has been an increasing impetus in developing more representative models of cancer that would reliably predict clinical activity of novel compounds in cancer patients.

Over the last five years, many advances have been made in culturing three-dimensional (3D) patient-derived tumor models [3–5]. The conditions used to grow these 3D tumor models were mostly modified from the conditions that were initially used to grow benign intestinal organoids from Lgr5+ intestinal stem cells [6]. These tumor models could faithfully recapitulate key characteristics of the original tumor, including histopathological, molecular and genetic features [3]. There have been extensive efforts to develop a 3D patient-derived tumor model for NSCLC. However, human lung 3D tumor model research is still in its infancy and most previous studies have focused on the development of lung organoids from human pluripotent stem cells [7]. Here, we sought to establish a method to culture 3D patient-derived tumor spheroids (PDS) for NSCLC that is amenable to drug screening and long term studies.

## 2 Materials and Methods

### 2.1 Human NSCLC tissues

NSCLC tissues were obtained from the Second Affiliated Hospital of Soochow University, China with written informed consent and approval by the Second Affiliated Hospital of Soochow University Ethical Committee of National Drug Clinical Trial Institution (S1 Fig). The institutional review board members are Liu Chunfeng, Wu Haorong, Zhang Quanying, Chen Weimin, Hong Xiaosu, Tian Ye, Dong Jixiang, Zhuang Zhixiang, Lu Qihua, Mao Qingyong and Chen Meijuan. All patients were diagnosed with stage I/II NSCLC and underwent curative video-assisted thoracoscopic surgery lobectomy. The surgically resected tumor was minced and digested overnight in 5 mg/ml collagenase type I (Thermo Scientific, Waltham, MA, USA) at 37°C. The dissociated cells were kept in complete medium containing 5% DMSO (Sigma, St. Louis, Missouri, USA) and stored in liquid nitrogen for future use. Human NSCLC cell line, H1299 was purchased from the Chinese Academy of Sciences Cell Bank (Shanghai, China).

### 2.2 3D cell culture

The cells were plated at 3 x 10^3^ cells/well in an ultra-low attachment 96-well plate (Corning, NYC, USA). Primary cells were cultured in Advanced DMEM/F-12 (Life Technologies, Waltham, MA, USA) supplemented with 1% FBS, 10% N-2 (Life Technologies, Waltham, MA, USA), 200ng/ml Noggin (PeproTech, Rocky Hill, NJ, USA), 1X B27 supplement (Life Technologies, Waltham, MA, USA, 1mM N-acetylcysteine and 100 U/ml penicillin and 100 μg/ml streptomycin while H1299 cells were cultured in RPMI 1640 medium supplemented with 10% FBS and 100 U/ml penicillin and 100 μg/ml streptomycin. The cells were maintained in humidified incubator with 5% CO_2_ at 37°C.

### 2.3 H&E staining

The tumor spheroids were processed for paraffin section using standard protocol. The tissues were fixed in 10% buffered formalin, paraffin embedded, and subsequently sectioned at 4μm for hematoxylin and eosin (H&E) staining. The slides were evaluated by a board-certified pathologist. All images were captured using an Olympus bx50 microscope at a total magnification of 100X.

### 2.4 Immunofluorescence staining

The tumor spheroids were fixed with 4% paraformaldehyde (PFA) for 30 min and permeabilized with 0.4% Triton X-100 for 50 min. The tissues were incubated with blocking buffer (PBS containing 2% BSA and 0.1% Triton X-100) for 1 h at room temperature. The primary antibodies, anti-Ki-67 (CST, Massachusetts, USA,1:800) and anti-Thyroid transcription factor-1 (CST, 1:50), were added and incubated at 4°C overnight. Alexa Fluor 488 (molecular probe, NYC, USA, 1:200) and Alexa Fluor 549 (Molecular Probes, 1:200) were used as secondary antibodies. Images of the tissues were captured using Nikon A1 confocal laser scanning microscope (Shinagawa, Tokyo, Japan) with 10X objective lens unless otherwise stated.

### 2.5 Drug treatment

Viability of the tumor spheroids following drug treatment was determined using CellTiter-Glo 3D cell viability assay (Promega, Madison, WI, USA). In brief, primary cells and H1299 cells were plated at 3000 cells/well (200μl/well) in an ultra-low attachment 96-well plate and allowed to form 3D multicellular tumor spheroids for 10 days before exposure to cisplatin (Jiangsu Hansoh pharmaceutical Group, China). The spheroids were then incubated with cisplatin for 72h at 37°C. At the end of incubation, equal volume of CellTiter-Glo 3D reagent were added and incubated for 30 min. The luminescence was measured using the Synergy™ HT Multi-Mode Microplate Reader (BioTek, Vermont, USA). All the tests were conducted in triplicates and standard deviations were reported.

## 3 Results and discussion

### 3.1 Development and optimization of NSCLC patient-derived tumor spheroids

**Fig 1:**
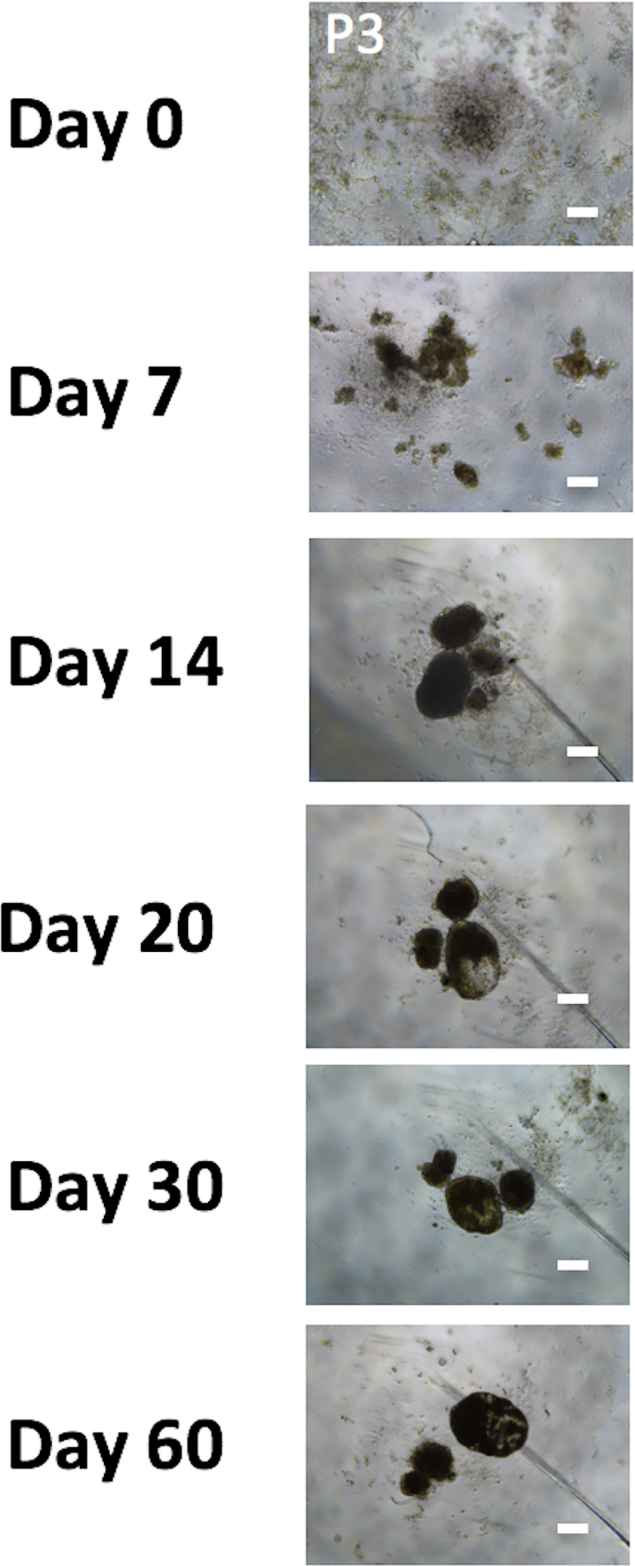
Establishment of patient-derived tumor spheroids over 120 days. Representative bright-field images of the tumor spheroids were shown and taken at x 40 total magnifications. Scale bars correspond to 200 microns.

Surgically resected tissue was obtained from chemotherapy-naive patients with stage I/II NSCLC. All samples were subjected to review by a board-certified pathologist to confirm diagnosis and assess the tumor cell content. Three PDS cultures from three tumor samples were successfully established and had been continuously cultured over 120 days (Fig 1). These tumor samples were previously frozen in liquid nitrogen and subsequently cultured directly to form PDS. The cell viability was > 90% upon thawing. The PDS reached the maximum spheroid size of about 500μm within 10 days of culture. All three PDS cultures could be readily expanded and frozen to create a biobank of PDS lines. Interestingly, the culture media used in this study didn't contain recombinant R-spondin1 or R-spondin1 conditioned media. However, it is important to note that the proliferation rate of the PDS was not very high with a subculture ratio of 1:2 every week. The addition of R-spondin1 might be able to increase the proliferation rate of PDS, which was shown to be critical to establish 3D organoid cultures from the human stomach, small intestine, colon, pancreas and liver [8]. The patient and tumor characteristics for the three established PDS can be found in S1 Table.

### 3.2 PDS recapitulates the cytological features and markers of NSCLC

**Fig 2:**
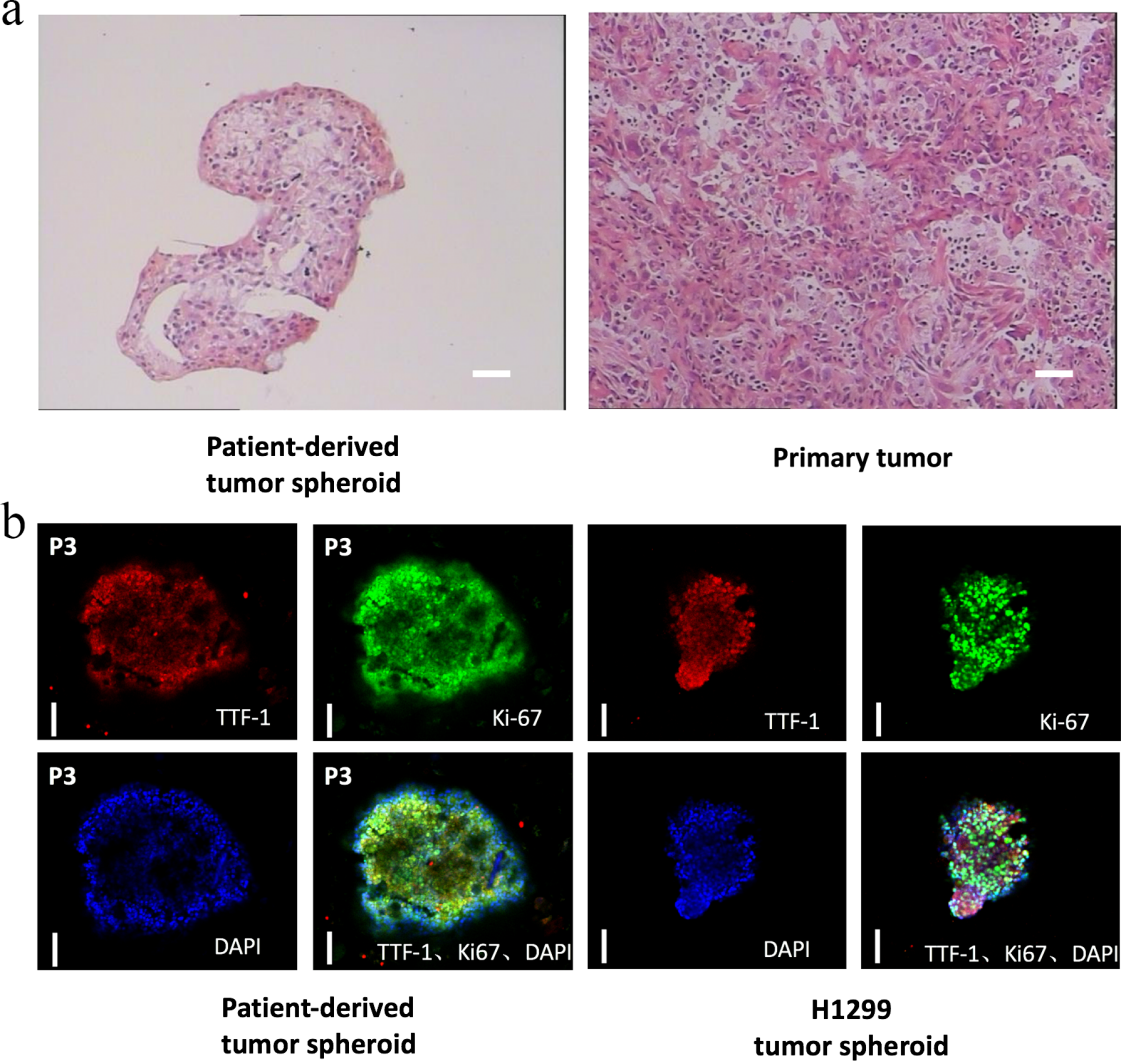
Patient-derived tumor spheroids maintain the cytological features and markers of the primary tumors. Representative images of the (a) H&E staining. scale bars correspond to 100 microns; (b) immunostaining of primary tumor, patient-derived tumor spheroid and/or H1299 tumor spheroid were shown. Scale bars correspond to 100 microns.

PDS showed tumor cells with high nuclear-cytoplasmic ratios, hyperchromatic nuclei with nuclear membrane irregularity, as seen in the matched patient tumor sample stained with H&E (Fig 2). Some tubular structures and branching morphogenesis were observed within the PDS under inverted microscope but further immunostaining of the structures is needed to confirm the findings (S2 Fig). All three PDS cultures stained positive for the lung tumor marker TTF-1, which confirmed the adenocarcinoma origin of the tumors (Fig 2). The role of TTF-1 in identifying primary lung adenocarcinoma from nonpulmonary, nonthyroid tumors has been well documented [9]. 3D H1299 tumor spheroids were established (S3 Fig) and stained positive for TTF-1 which had been previously reported (Fig 2) [10]. Positive staining for Ki-67 also indicated that the PDS retained their proliferation potency. H&E and immunostaining of multiple PDS passages also revealed that tumor cells’ cytological features remained stable and the clinical markers consistently retained with serial passaging (data not shown).

### 3.3 PDS is amenable to drug screening

**Fig 3:**
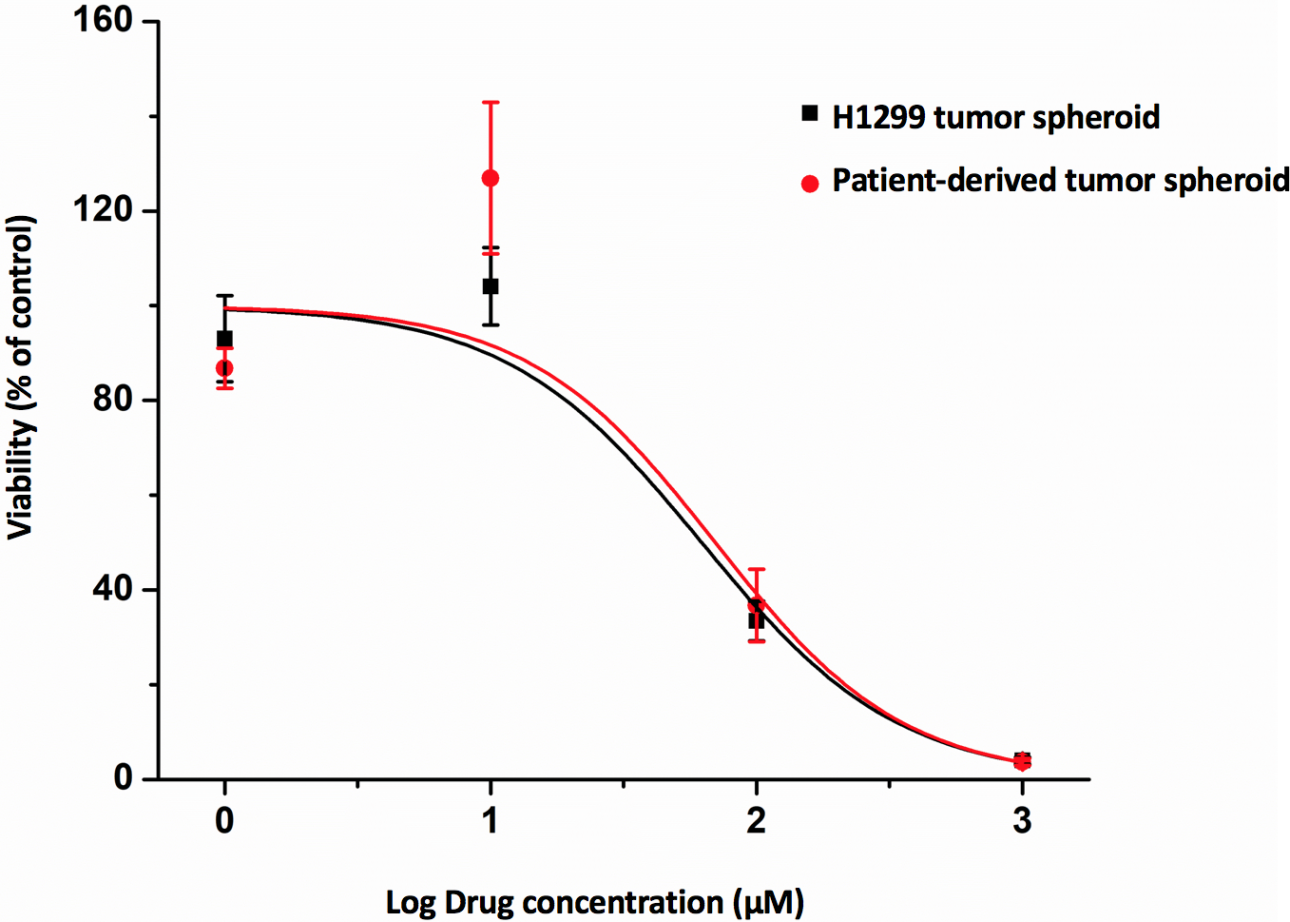
Cytotoxicity of cisplatin in patient-derived tumor spheroids and H1299 tumor spheroids. Cell viability was assessed using CellTiter-Glo 3D Cell Viability Assay after 72h of drug exposure. Data are mean ± S.D. obtained from one independent experiment done in triplicates.

To demonstrate the utility of PDS as a drug screening platform, the cytotoxicity of cisplatin in PDS and H1299 tumor spheroids was investigated using CellTiter-Glo 3D Cell Viability Assay. The concentration of cisplatin tested ranged from 1–1000 μM with an exposure period of 72h. Inhibitory concentration at 50% effect level (IC_50_) was estimated using the OriginPro software. The results showed a dose-dependent decrease in cell viability after exposure to cisplatin for 72 h, with an IC_50_ value of 70.0μM and 62.2μM for PDS and H1299 tumor spheroids, respectively. The IC_50_ value was much higher than the IC_50_ values reported in most 2D NSCLC cell lines, which are generally <10μM unless in inherent and acquired cisplatin-resistant NSCLC cell lines [11]. The phenomena of increased IC_50_ values in 3D cultures as compared to conventional 2D monolayer cultures has been well documented [12]. However, the difference in cisplatin cytotoxicity between the PDS and H1299 tumor spheroid was difficult to discern. (Fig 3, for raw data, please refer to S2 Table) We reasoned that the PDS derived from early-stage, chemo-naive NSCLC patients might not carry any aberrations that might affect the activity of cisplatin. A larger drug panel might be needed to establish the superiority of PDS over tumor spheroids of cell lines. Nevertheless, it is envisioned that the PDS could be used to demonstrate the correlation between the IC_50_ of the PDS and clinical chemotherapy response of the individual patient, which was not done in this study as the patients did not undergo any chemotherapy.

The objective of our study is to establish 3D PDS models in order to conduct *in vitro* studies in parallel with clinical patient drug treatment in future. The long term *in vitro* culture is the main objective of this study. The next step of our study is to establish drug resistant model in order to study the drug resistant mechanism and provide insights for medical doctors.

In this study, we also showed that the PDS retains the phenotypic cellular characteristics of the original tumor after all this time in culture. The H&E staining results from patients’ tissue and our cell spheroids after 120-day culture were compared (Fig 2A). There is no obvious difference between these two groups of samples. In future, more in-depth characterization data will be done in order to have a comprehensive evaluation of our novel model. The genetic test and more immunostaining studies will be conducted to have a more in-depth evaluation of this novel platform.

Compared with 2D models, this 3D PDS models can better represent the real interaction between drugs and cancer tissues. This also applies to some cutting-edge biotechnologies such as the use of oncolytic viruses. Firstly, the spheroids contain not only cancer cells but also normal cells such as fibroblast. This model can be used to study the targeting effect of drugs or oncolytic viruses to cancer cells. Moreover, the spheroids are excellent 3D models to study the penetration of drugs or virus to solid tumor. Last but not the least, our current technologies allow the patient-derived spheroid to be cultured *in vitro* for more than 120 days. This allows the long term and in-depth study of the mechanisms.

## 4 Concluding remarks

Collectively, our current study has established the long-term culture conditions of PDS for patients with NSCLC that allow recapitulation of cytological features and markers of the primary tumor, and demonstrated the drug screening utility of PDS. Further studies of PDS involve comprehensive characterization of the gene expression profiles and mutations present in the patient-derived tumor spheroid to demonstrate that the model could also recapitulate the molecular and genetic diversity of NSCLC tumor. In future, we will establish a biobank with cells from Asian NSCLC samples. These cells contain the heterogenetic information of lung cancer cells from Asian patients, which is critical for the studies of the Asian-patient specific NSCLC [13–14].

## Supporting information

S1 Table Patient and tumor characteristics. (DOCX)

S2 Table Raw data for Fig 3. (XLSX)

S1 Fig The consent and approval by the Second Affiliated Hospital of Soochow University Ethical Committee of National Drug Clinical Trial Institution. The institutional review board members are Liu Chunfeng, Wu Haorong, Zhang Quanying, Chen Weimin, Hong Xiaosu, Tian Ye, Dong Jixiang, Zhuang Zhixiang, Lu Qihua, Mao Qingyong and Chen Meijuan.
(TIFF)

S2 Fig Representative bright-field image of the patient-derived tumor spheroid at day 60 was shown. The image was taken at x 100 total magnification. Scale bar corresponds to 100 microns.
(TIFF)

S3 Fig Establishment of H1299 tumor spheroids over 30 days. Representative bright-field images of the tumor spheroids were shown and taken at x 40 total magnifications. Scale bars correspond to 200 microns.
(TIFF)

## Abbreviations

EGFR: epidermal growth factor receptor
H&E: hematoxylin and eosin
NSCLC: non-small cell lung cancer
PDS: patient derived tumor spheroids
TNM: tumor, node and metastasis
