# Supplementary material for: Establishment of patient-derived tumor spheroids for non-small cell lung cancer
Source: PLoS One. 2018 Mar 15;13(3):e0194016. doi: 10.1371/journal.pone.0194016 (PMC5854348; doi:10.1371/journal.pone.0194016)
Supplement: S1 Table — (DOCX) [file pone.0194016.s001.docx]

**S1 Table.** Patient and tumor characteristics.

| Sample | Gender | Age at diagnosis | TNM classification | EGFR status | CK7 | Ki67 |
| --- | --- | --- | --- | --- | --- | --- |
| P1 | Male | 53 | T2bN0M0 | negative | + | + |
| P2 | Male | 70 | T1cN0M0 | negative | + | + |
| P3 | Male | 75 | T1bN0M0 | negative | + | + |
