# Supplementary figures and images for: Establishment of patient-derived tumor spheroids for non-small cell lung cancer
Source: PLoS One. 2018 Mar 15;13(3):e0194016. doi: 10.1371/journal.pone.0194016 (PMC5854348; doi:10.1371/journal.pone.0194016)

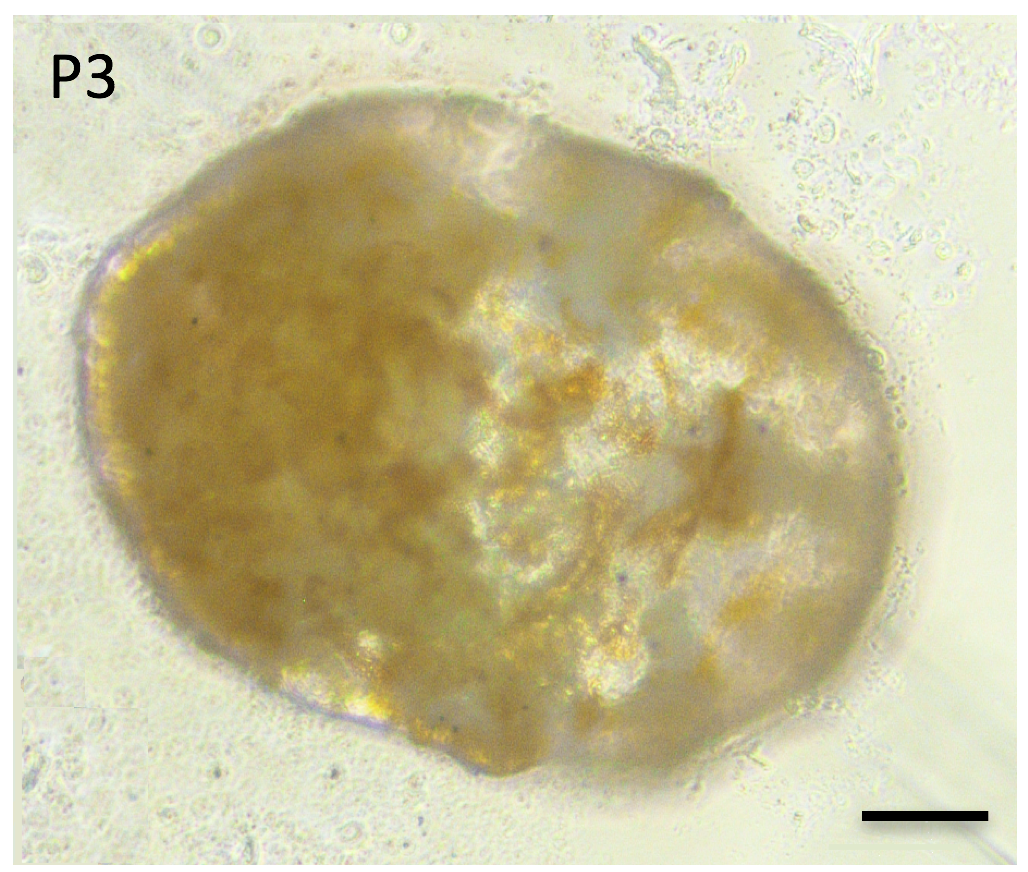

Supplement: S2 Fig — The image was taken at x 100 total magnification. Scale bar corresponds to 100 microns. (TIFF) [file pone.0194016.s004.tiff]

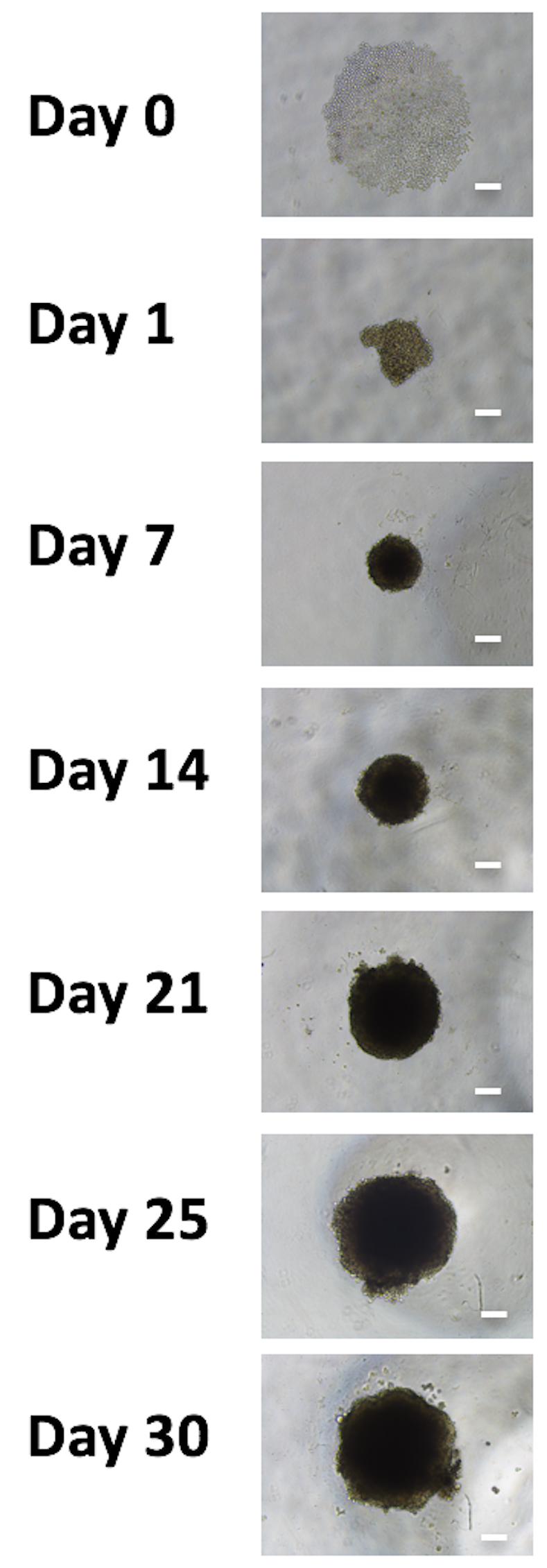

Supplement: S3 Fig — Representative bright-field images of the tumor spheroids were shown and taken at x 40 total magnifications. Scale bars correspond to 200 microns. (TIFF) [file pone.0194016.s005.tiff]
